# A Killer Immunoglobulin - Like Receptor Gene - Content Haplotype and A Cognate Human Leukocyte Antigen Ligand are Associated with Autism

**DOI:** 10.4172/2165-7890.1000171

**Published:** 2016-03-28

**Authors:** Anthony Torres, Jonna Westover, Michael Benson, Randall Johnson, Annelise Dykes

**Affiliations:** Center for Persons with Disabilities, Utah State University, Logan, Utah, USA

**Keywords:** Autism, Killer - cell immunoglobulin - like receptors (KIR), Human leukocyte antigen (HLA) alleles, Transmission disequilibrium test (TDT), KIR gene

## Abstract

The killing activity of natural killer cells is largely regulated by the binding of class I human leukocyte antigen cognate ligands to killer cell immunoglobulin - like receptor proteins. The killer cell immunoglobulin - like receptor gene - complex contains genes that activate and others that inhibit the killing state of natural killer cells depending on the binding of specific human leukocyte antigen cognate ligands. It has been suggested in previous publications that activating human leukocyte antigen/killer - cell immunoglobulin - like receptor complexes are increased in people with autism. We present data, which suggests that an activating cB01/tA01 killer cell immunoglobulin - like receptor gene - content haplotype and the cognate ligand human leukocyte antigen - C1_k_ that activates this haplotype is significantly increased in autism. This is an important observation suggesting that the interaction between two proteins encoded on different chromosomes increases natural killer cell killing in autism.

## Introduction

The etiology of autism is not well understood despite extensive research over the last 4 decades. Autism is a complex neurodevelopmental disorder characterized by deficits in communications, social skills, and the presence of restrictive and repetitive stereotyped behaviors. Several studies have noted a strong familial clustering suggesting that heredity is of major importance and that about 50% of genetic risk should be found in common genetic variants [1–4]. However, only a small number of autism cases can be associated with specific genes using modern typing technologies and the majority of genetic polymorphisms are rare variants and not common variants [4–9].

One interpretation is that rare variants are major contributors to genetic diseases and autism involves the interaction of many rare variants, especially in the brain [10,11]. However, when examining whole - genome sequence data of autism quartet families (two autism affected siblings and parents), a surprising observation suggested that the majority (69%) of autism affected siblings carried different rare variant mutations [12]. Another interpretation is that common variants are not clearly detected by these screening methods. It has become obvious that “autism genetics” is far more complicated than previously thought. Our research in autism has suggested that complex genetic structures like HLA haplotypes are important in understanding the genetics of autism, and genome sequencing and microarray SNP data do not conclusively define complex genetic structures like large multigene haplotypes [13–16].

Evidence has been mounting over the years that indicate immune involvement in the etiology of certain cases of autism [17–20]. Autism immune associations include maternal humoral antibodies against fetal brain proteins, which correlate with autoimmunity in mothers of children with autism, and elevated proinflammatory cytokine levels, which have been observed in the serum and in the brain of children with autism [21–24]. Additionally, there is considerable evidence from association and linkage studies that suggests the involvement of HLA alleles/haplotypes in the etiology of autism [8,13,14,25–27]. Although the HLA complex (chromosome 6) is considered to be the most important region for immune function genes, there are other important immune complexes like the leukocyte receptor complex (LRC) on chromosome 19, which contain at least six families of genes encoding immunoglobulin superfamily receptors interspersed with other loci [28].

**Figure 1:**
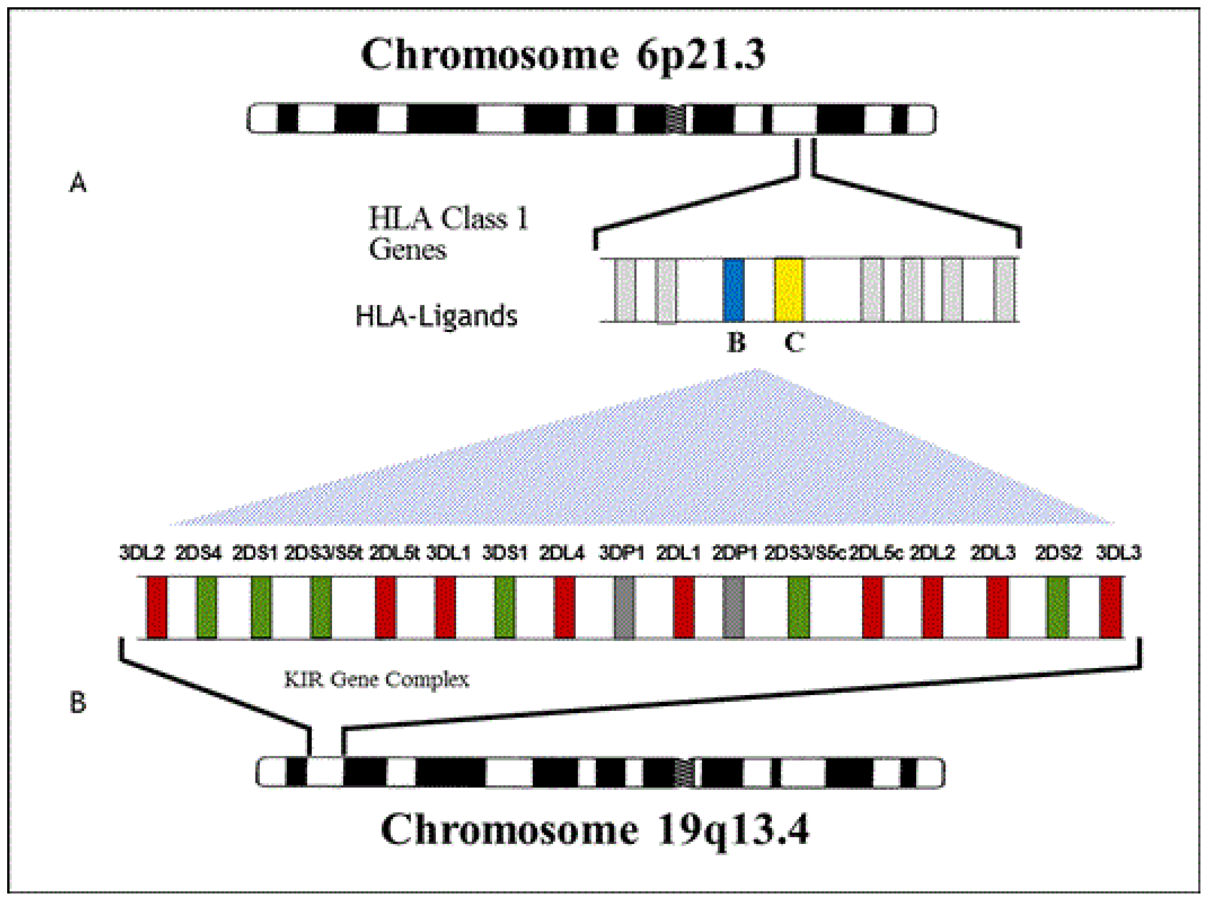
location of HLA ligands on human chromosome 6 and the KIR gene complex on chromosome 19

One of the most studied gene families in the LRC, the killer - cell immunoglobulin - like receptor (KIR) complex, encodes receptor proteins that help enable natural killer lymphocytes (NK - cell) to survey somatic cell surfaces for the presences of HLA class I protein ligands. NK - cell killing is initiated when KIR receptors do not detect certain HLA class I proteins on the cell surface, often as a result of viral infection or a malignancy [29]. Specific KIR genes encode proteins that activate NK - cell killing while others inhibit killing, thus the terms activating or inhibiting genes. There are significant differences in the inherited ratios of the activating/inhibiting KIR genes in individuals, and imbalances in the ratios of KIR inhibiting/activating genes have been associated with several autoimmune diseases, malignancies, infections, and abnormal pregnancy [30]. Subjects with autism have an increased frequency of activating KIR genes along with very specific cognate HLA ligands that interact with activating or inhibitory genes [31,32]. Figure 1 demonstrates the location of HLA ligands on human chromosome 6 and the KIR gene complex on chromosome 19. KIR haplotypes are composed of different combinations of these inhibitory and activing genes, hence the term gene - content haplotype [33].

We present data that suggest an association between autism and KIR gene - content centromeric/telomeric haplotypes. In addition, there are fewer HLA: KIR inhibitory interactions between the HLA ligands and KIR gene - content (centromeric/telomeric) haplotypes in the autism population. In summary, these observations made with a relatively small population of 89 AGRE families suggest that KIR gene - content haplotypes and their HLA cognate ligands may be important in the understanding of autism genetics.

## Materials and Methods

The KIR haplotyping of the 89 Autism Genetics Resource Exchange (AGRE) Caucasian subjects (73 males and 16 females) required about 300 ng of genomic DNA or whole genome amplified DNA to generate 14 large amplicons each containing several contiguous KIR genes [33]. This PCR dye - termination sequencing procedure delineates the gene copy number for all 16 KIR genes by clearly identifying activating/inhibitory KIR genes that have very similar DNA sequences. The KIR genes also have alleles that are not addressed at this time.

Genomic DNA was found to type better than whole genome amplified DNA for haplotype determination: presumably, whole - genome amplified DNA has smaller amplicons that do not contain all the KIR genes to obtain a clear gene copy number. The 89 AGRE samples were typed at Sisco Genetics, Seattle, Washington, by the PCR dye - terminating sequencing procedure to determine the KIR gene copy numbers. The same subjects were previously KIR genotyped and the KIR genotype and KIR gene copy numbers were compared to demonstrate the importance of KIR gene copy numbers (Table 1) [32].

Genomic DNA (about 10 ng) from AGRE subjects and parents were genotyped for HLA - C1_k_/C2_k_ and Bw4 alleles using high - resolution melt in an Applied Biosystems 7500 Fast Real Time PCR System instrument. A polymerase chain reaction (PCR) assay with site - specific primers (SSP) for HLA - C1_k_ and C2_k_ alleles was used with excellent results [34]. The same high - resolution melt system was used to genotype Bw4 and non - Bw4 alleles with primers specific for HLA - A and primers specific for HLA - B [35,36]. The KIR gene - copy number and KIR gene - content haplotypes of a Caucasian population of 4,512 subjects was used as the control population for statistical comparisons [33].

The 89 AGRE subjects and their parents were typed for HLA - class I (A,B,C) allotypes using commercial PCR typing kits following the manufacturer’s instructions (SSP - Unitray Kit, Invitrogen, Camarillo, CA). All HLA - A&B peptide - binding alleles are either Bw4 or Bw6 serological alleles. The following HLA - B alleles have the Bw4 KIR binding epitope: B5, B13, B17, B27, B37, B38, B44, B47, B49, B51, B52, B53, B57, B58, B59, B63, and B77. The Bw6 epitope does not bind to KIR receptors and is not discussed further in this paper. All peptide binding HLA - C alleles are either HLA - C1_k_ (C^*^01, 03, 07, 08, 12, 14, 16) or HLA - C2_k_ (C^*^02, 04, 05, 06, 15, 17, 18) KIR binding alleles. The genotyping of the Bw4, non - Bw4, HLA - C1_k_ and C2_k_ alleles was in excellent agreement with serological alleles determined from HLA - class I (A, B, C) allotyping. The inherited and noninherited Bw4, C1_k_, and C2_k_ alleles were compared for activation/inhibition with KIR telomeric/centromeric haplotypes. The noninherited Bw4, C1_k_, and C2_k_ HLA alleles served as controls, as they are not associated with autism.

Odds ratios (OR) and p values were calculated by multiple logistic regression for all KIR genes and KIR gene - content haplotypes with the significance level set at α = 0.05. The KIR gene copy number and the five centromeric and telomeric haplotypes of the 89 AGRE autism subjects were compared with the Caucasian control population of 4,512 subjects (9,024 chromosomes or haplotypes) [33]. The Transmission Disequilibrium Test (TDT) was performed using the binomial distribution and p values for the TDT test are two - tailed [37].

## Results

Although research associating activating KIR genes with autism is very informative, it is incomplete, as KIR genotyping does not identify the gene copy number necessary for accurate haplotype determination. KIR gene copy number can be clearly determined using a new sequencing method [33]. As shown in Table 1, the KIR genotype is compared against the gene copy number of the same 89 subjects by dividing the GCN by the genotype. There are eight genes that have a GCN/genotype ratio of about 1, which means that there is a single gene present on one of the two chromosomes. There are four KIR genes with GCN/genotype ratios of about 2, meaning there are two copies of the gene, or one present on each chromosome. These four KIR genes are considered structural genes, as they are present in all chromosomes. Five KIR genes have GCN/genotype ratios between 1 and 2, meaning that some subjects have a gene on only one chromosome and others have two gene copies, or one on each chromosome. Seven subjects have three copies of 3DP1 on the centromere side and three copies of 2DL4 on the telomere side. Gómez - Lozano et al. noted that about 4.5% of the Caucasian population have these duplications [38]. The gene copy number for pooled activating and inhibitory KIR genes of autism subjects versus control subjects suggests a significant increase in activating genes in autism subjects (p = 0.0029; Table 2). These results are in agreement with previous publications, which suggested an increase in activating genes as determined by genotyping [31,32].

**Figure 2:**
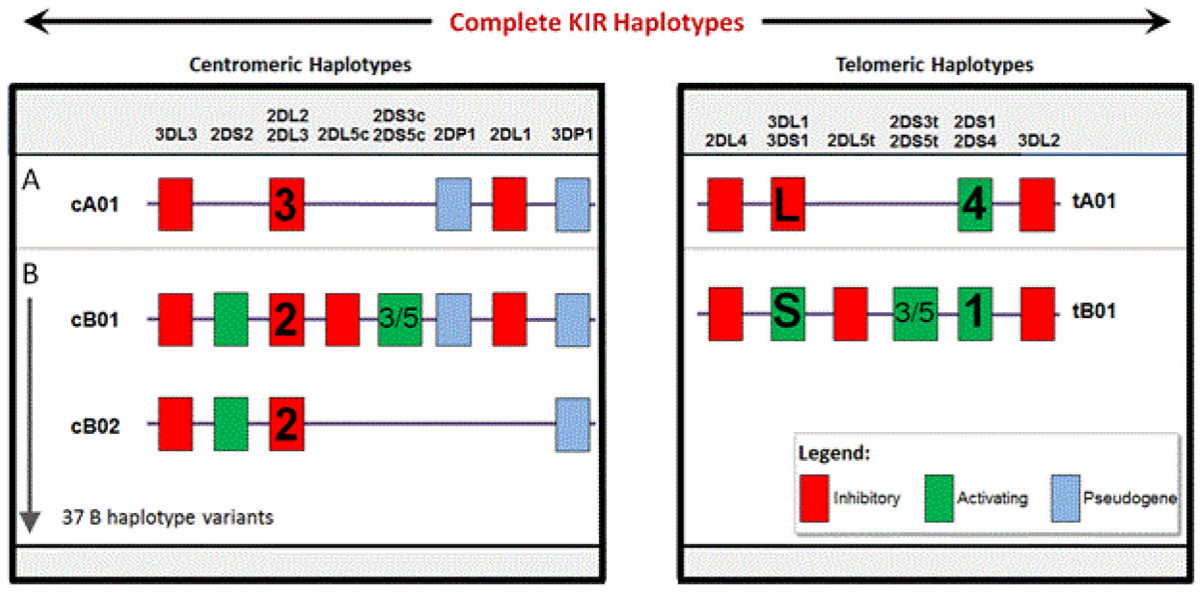
Complete KIR gene haplotypes

The accurate GCN allows for the construction of individual KIR gene - content haplotypes. In Figure 2 we show three centromeric haplotypes (cA01, cB01, and cB02) and two telomeric haplotypes (tA01 and tB01) based on the GCN data of each individual. Complete KIR gene - content haplotypes encompasses up to 16 genes and 2 pseudo genes in about 150,000 bp [38].

In the AGRE samples, two centromeric KIR gene - content haplotypes are statistically significant: cA01 (p = 0.00001; OR = 0.53) and cBO1 (p = 0.0045; OR = 1.69) (Table 3). The telomeric KIR gene - content haplotype tA01 is significantly decreased (p = 0.0004; OR = 0.53) in the subjects with autism compared to control subjects. Two of the three haplotypes that reached statistical significance were decreased in frequencies (cA01, p = 0.00001, odds ratio 0.53 and tA01, p = 0.00004, odds ratio 0.53) from control haplotypes. The centromeric haplotype cB01 was increased over control frequencies (p = 0.0045, odds ratio 1.69) (Table 3).

Complete KIR gene - content haplotypes have a centromeric haplotype and a telomeric haplotype meaning there are 6 possible complete KIR haplotypes in this population (Table 4). We assembled complete KIR gene - content haplotypes for 170 out of 178 possible haplotypes. The only KIR gene - content haplotype to reach statistical significance is cB01/tA01 (p = 0.002; OR = 2.06).

The strong familial clustering of autism has been much of the driving force behind the search for autism - associated genes [3]; however, genomic screening methods have not been very successful in finding common genetic variants. Previously we have published data utilizing the transmission disequilibrium test (TDT), which suggested genetic linkage of common variant HLA alleles and HLA extended haplotypes with autism [13,14]. In the current application, the TDT data were approaching significance for HLA - C1_k_ and the 2DL2 and 2DS2 genes (p = 0.1114, and p = 0.2026, respectively); however, only 49% of the parents were informative for HLA - C group transmission as much of the data could not be used due to allelic homozygosity.

In this paper, we present data that suggest genetic association of HLA ligands with KIR receptors. As shown in Table 5, there are only two ligands in all of the HLA - C peptide binding alleles: HLA - C1_k_ has an asparagine in amino acid (aa) position #80, and HLA - C2_k_ has a lysine in aa position #80. All HLA - B alleles have HLA - Bw4 or HLA - Bw6 (Bw4 or Bw6) serological epitopes. The Bw4 epitope contained in amino acids position 77 – 83 of the HLA - B heavy chain is a KIR ligand for certain telomeric KIR receptors (Table 5).

The HLA - Bw4 ligand (Bw4; p = 0.3764) (data not shown) and C1_k_ and C2_k_ alleles (p = 0.1378 and p = 0.1378) were not significant when compared to the noninherited parental alleles. However, when examining KIR ligand/KIR haplotype associations, subjects with a cB01 haplotype and two C1_k_ ligands was significant (p = 0.026, OR = 2.941) (Table 6).

The interaction of HLA ligands with KIR receptors generates diversity depending on the gene copy number of HLA - C alleles [39] and the KIR gene - content haplotype. Complicated interactions between HLA and KIR can occur. For example, C1_k_ can inhibit certain KIR genes and activate others (Table 5). In addition, individuals with one C1_k_ and one C2_k_ have the potential for two inhibitory HLA: KIR interactions while individuals homozygous for the C1_k_ or C2_k_ ligands have only the potential for one inhibitory HLA: KIR interaction. When comparing HLA inherited with noninherited C1_k_ and C2_k_ there is a suggestion that subjects with C1_k_/C1_k_ and a cB01 haplotype would not only have more activating genes but also fewer inhibitory interactions (Table 6). It has been reported that HLA: KIR ligands are not associated with KIR genes in the general population [39]; however, in the AGRE autism subjects examined in this project, our data suggest an association between the HLA - C1_k_ and the cB01 KIR gene - content haplotype (Table 6).

## Discussion

NK - cells are a first line of protection in innate immunity and respond to infection without prior sensitization by producing an inflammation response to help control the microbial invasion. NK - cells exert this effect by physically interacting with somatic cells and producing cytokines to recruit other immune cells. This physical interaction can lead to the killing of abnormal cells lacking HLA class I molecules usually due to infection or malignancy. The killing process is a very complex interaction involving six main receptor complexes in the LCR, including the

Inhibitory/activating genes in the KIR complex and HLA class I molecules. It is rather interesting that HLA ligands encoded on chromosome 6 can bind either activating or inhibitory KIR receptors expressed on NK - cells depending on the amino acid in position #80 of the HLA beta chain [40]. For example, an asparagine in position #80 (C1_k_) is a ligand for 2DS2 and 2DL2; whereas, a lysine in position #80 (C2_k_) is a ligand for 2DS1 and 2DL1. The same specificity occurs on the receptor end: a lysine in position #44 is on the 2DS2 and 2DL2 KIR proteins; whereas, a methionine in position #44 is on the 2DS1 and 2DL1 KIR proteins. Single DNA base differences in HLA - C ligand genes encode different amino acids and the different amino acids are ligands for different KIR receptors (below).

HLA - C1_k_ aa #80 ASN (codon #AAC) - - - - - - - - - - - - - - KIR - 2DS2 and KIR - 2DL2
HLA - C2_k_ aa #80 LYS (codon #AAA) - - - - - - - - - - - - - - KIR - 2DS1 and KIR - 2DL1

A genetic haplotype is a contiguous combination of DNA sequences including single nucleotide polymorphisms (SNPs) that are inherited on a single chromosome. They can be short (10 – 100 base pairs) or very long (millions of base pairs) containing many genes. Haplotype structure is often essential for the understanding of allele - specific events such as protein structure, methylation, outcomes in transplantation, and disease prediction [41]. However, haplotype structure is often difficult to determine from microarray and DNA sequence as one cannot easily assign a DNA sequence to a particular chromosome [15]. This means that most genetic data, whether short tandem repeats, SNPs, or CNV polymorphisms do not readily allow for the haplotype structure on the two chromosomes. Numerous methods to determine haploid sequences have been accomplished by tedious examination of linkage disequilibrium maps, typing single sperm cells, comparing long - range sequences between parents and subjects, and single molecule DNA typing [42–46].

We have published numerous papers over the last 30 years concerning the association and linkage of HLA alleles and HLA extended haplotypes with autism [13]. Autism associations were determined by chi - square analysis and linkage was determined by transmission disequilibrium test (TDT) analysis using a familial design [32]. These large 4.5 million base pair extended haplotypes contain about 150 genes in class I, II, and III regions and are rather easy to identify by standard typing techniques. Extended HLA haplotypes have also been observed in subjects with autism from Saudi Arabia [25]. Application of the TDT test to autism genetic sequencing studies has been recently applied to identify haplotypes outside of the immune system [16,47]. Pan et al. used a group - wise TDT instead of a familial - based design as another way to examine genetic haplotypes in autism [16].

Attempts to accurately delineate KIR haplotypes have been especially difficult due to extreme complexity in the KIR gene structure, including remarkably similar DNA sequences of inhibitory and activating genes that are absent or present in different haplotypes. In addition, because of this mixture of genes on two different chromosomes, KIR gene copy numbers have been difficult to determine. Two basic haplotypes have been defined on the basis of gene content, and are termed haplotypes A and B. Haplotype A is uniform in terms of gene content and is composed of five inhibitory genes (KIR2DL1, 2DL3, 3DL1, 3DL2, and 3DL3) and one activating gene KIR2DS4. The B group of activating haplotypes is defined as having one or more of the following: KIR2DL5, 2DS1, 2DS2, 2DS3, 2DS5 and 3DS1.

About 50% of KIR haplotypes are of the A type meaning that about 25% of subjects have two A haplotypes. It has, therefore, been easy to identify subjects with two inhibitory (AA) KIR haplotypes from genotyping data as the only activating gene present is 2DS4 (one on each chromosome). Type B haplotypes are referred to as activating haplotypes as they contain more activating KIR genes; however, they cannot be positively determined without an accurate gene copy number as certain genes can be present on 1 or 2 chromosomes. Until recently, it could not be accurately determined if a subject had one or two activating B haplotypes [29]. Research in the Geraghty laboratory made a significant contribution in understanding the KIR haplotype structure by enabling the determination of accurate GCN for each KIR gene. Their approach of placing KIR genes into smaller centromeric and telomeric haplotypes allows one to connect a centromeric with a telomeric end for a complete 16 - gene KIR haplotype [33].

It has been shown over the last several years that imbalances in the inhibitory/activating KIR receptors on NK - cells and cognate HLA proteins are present in numerous autoimmune diseases such as psoriasis, scleroderma, rheumatoid vasculitis, as well as several cancers including cervical cancer, nasopharyngeal carcinoma, and most recently autism [30–32].

Warren noted an increase in NK - cell count in autism along with decreased NK - cell killing upon stimulation. Enstrom et al. confirmed these results and made an important contribution by demonstrating that resting NK - cells from subjects with autism had an increased killing response [48]. This increased baseline NK - killing response suggested to us that perhaps subjects with autism have an increase in activating KIR genes as well as a decrease in HLA: KIR inhibitory interactions.

Our present data suggest an important interaction in proteins encoded in two different immune gene complexes on two different chromosomes in autism. The conclusions in this research offer a partial explanation for the observation that NK - cells from autism subjects have a higher resting killing rate than in control subjects [48,49]. We were unable to show linkage of the HLA (KIR) ligands to autism in this small sample; however, the statistics suggest interesting trends in this population that will require a larger sample to establish linkage with statistical rigor.

One must remember that the proteins encoded in the HLA and KIR haplotypes communicate with each other in a very specific manner to enhance or suppress NK - cell killing. The full meaning of these observations is unknown at this time; however, it should be mentioned that both of these regions contain common genetic variants (5% greater or less than control frequencies), which have not been identified by genomic screening techniques. It is easy to understand how genetic screening methods miss interactions that occur by single bases on two different chromosomes. We have published on the increase of the HLA - A2 allele in autism [13]. Examination of the HLA region in the 89 AGRE subjects against noninherited allele controls suggests that HLA - A2 allele is also increased (*p* = 0.022; *OR* = 1.74). This may be important as the class I alleles present peptides to CD8 + T - cells to boost cellular immunity. This suggests that innate and cellular immunity may be occurring in the same AGRE subjects. A more complete examination of HLA alleles/haplotypes in these subjects will be reported separately.

Other genes on the LRC on chromosome 19 such as ILT and LAIR, and genes such as NKG2D and NK2D in the Natural Killer Gene Complex (NKC) on chromosome 12 also influence NK - cell killing through HLA interactions. More research is needed in these areas to better understand complex multi - chromosomal interactions and to determine KIR gene - content haplotype inheritance and possible linkage in autism. Last, elevated NK - cell killing and cytokine production could be important in the placenta and in early brain development.

It may be possible to develop immune treatments as HLA: KIR interactions are well understood. For example, monoclonal antibodies targeted to either HLA or KIR epitopes may be useful in blocking unwanted responses. Also, soluble protein decoys to block or enhance HLA and KIR binding sites could be used to modify the immune reponse in certain subjects [50,51].

## Conclusions

The data presented in this paper suggest that an activating KIR gene - content haplotype cB01/tA01 is increased in autism and that the cognate HLA ligand (HLA - C1_k_) that activates an activating gene (2DS2) and inhibits two inhibitory genes (2DL2 & 2DL3) (Table 5) is also increased in the subjects with this haplotype. A more complete understanding of the interactions on these immune proteins encoded on two separate chromosomes may make it possible to use specific monoclonal antibodies and/or HLA/KIR decoy peptides in treatment.

## Figures and Tables

**Table 1:** Comparison of KIR Genotype and KIR Gene Copy Number in 89 AGRE Subjects. Note: KIR genotyping only determines if a gene is absent or present; whereas, the GCN determines the number of specific KIR genes in a diploid individual. By dividing the GCN for each KIR gene by the genotype a gene content ratio is obtained.

**KIR gene**	**AGRE genotype**		**AGRE gene copy number (GCN)**					**GCN/genotype ratio**
	**Present**	**Absent**	**0**	**1**	**2**	**3**	**Total**	61/56 = 1.09
2DS2	56	33	33	51	5	0	61	
2DL2	53	36	36	50	3	0	56	56/53 = 1.06
2DL3	77	12	12	45	32	0	109	109/77 = 1.42
2DL5c	27	62	62	25	2	0	29	29/27 = 1.07
2DS35c	27	62	62	25	2	0	29	29/27 = 1.07
2DP1	88	1	1	29	59	0	147	147/88 = 1.67
2DL1	88	1	1	26	62	0	150	150/88 = 1.70
3DP1	89	0	0	4	78	7	181	181/89 = 2.03
2DL4	89	0	0	4	78	7	181	181/89 = 2.03
3DL1	85	4	4	39	45	1	132	132/85 = 1.55
3DS1	42	47	47	35	7	0	49	49/42 = 1.17
2DL5t	44	45	45	40	4	0	48	48/44 = 1.09
2DS35t	44	45	45	40	4	0	48	48/44 = 1.09

**Table 2:** The Average Number of KIR Activating (S) and Inhibitory (L) Genes in AGRE Subjects Compared to Average Number of (S) and (L) Genes in the Control Population [33]

KIR genes	Average activating (S) genes/AGRE subject (n = 178)	Average inhibitory (L) genes/Control population (n = 9,024)	p value	OR
2DS2, 2DS3/5c, 3DS1, 2DS3/5t, 2DS1, 2DS4	2.14	1.87	0.0029	1.19
3DL3, 2DL2, 2DL5c, 2DL1, 2DL4, 3DL1, 2DL5t, 3DL2, 2DL3	6.04	5.93	0.038	1.25

**Table 3:** Centromeric and Telomeric KIR Haplotypes Constructed From Individual GCNs Were Compared to 9024 KIR Normal Control Gene - Content Haplotype

Haplotypes	Control	Autism	Fisher’s exact P	OR	95% confidence interval
	(n = 9,024)	(n = 178)			
Centromeric					
cA01	6,248	110	0.00001	0.53	(0.40, 0.70)
cB01	1,184	41	0.0045	1.69	(1.19, 2.39)
cB02	1,566	27	0.1576	0.73	(0.49, 1.11)
cB03	26	0	1	-	-
Telomeric					
tA01	6,968	130	0.00004	0.53	(0.40, 0.71)
tB01	2,055	48	0.7347	1.06	(0.76, 1.47)

**Table 4:** Complete KIR Haplotypes of AGRE Subjects versus Caucasian Controls [33]. Eight AGRE haplotypes could not be determined from the 178 possible haplotypes.

KIR haplotypes	AGRE	Control	p value	Odds ratio
	(n = 170)	(n = 9,024)		
cA01/tA01	84	5,133	0.06	0.74
cA01/tB01	23	1,114	0.638	1.11
cB01/tB01	13	461	0.157	1.538
cB02/tA01	17	1,112	0.41	0.791
cB01/tA01	25	697	0.002	2.06
cB02/tB01	8	480	0.863	0.879

**Table 5:** HLA Ligand Activation or Inhibition of KIR Haplotypes.

KIR Haplotypes	HLA ligands
cA01^a^	C1_k_ Inhibits (2DL3), C2_k_ Inhibits (2DL1)
cB01	C1_k_ Inhibits (2DL2), C1_k_ Activates (2DS2), C2_k_ Inhibits (2DL1)
cB02	C1_k_ Inhibits (2DL2), C1_k_ Activates (2DS2)
tA01^b^	Bw4 Inhibits (3DL1),
tB01	Bw4 Activates (3DS1), C2_k_ Activates (2DS1)

a Centromeric haplotypes - - Bw4 is not a ligand and

b Telomeric haplotypes - - C1_k_ is not a ligand.

**Table 6:** Inherited and Noninherited HLA - C Ligand Binding to the KIR Centromeric Haplotype cB01. 1 HLA - C1_k_ (C*01, 03, 07, 08, 12, 14, 16) and 2 HLA - C2_k_ (C*02, 04, 05, 06, 15, 17, 18) allotypes.

Inherited HLA KIR ligands determined from HLA allotyping and genotyping			Noninherited HLA Ligands from HLA allotyping		KIR - HLA associations	
Haplotype	HLA ligand	%	HLA ligand1,2	%	p value	OR
Centromeric	(42 subjects)		(39 subjects)			
cB01	C1_k_/C1_k_ (25)	59.5	C1_k_/C1_k_ (13)	33.3	0.026	2.941
cB01	C1_k_/C2_k_ (15)	35.7	C1_k_/C2_k_ (20)	51.3	0.183	0.528
cB01	C2_k_/C2_k_ (2)	4.8	C2_k_/C2_k_ (6)	15.4	0.146	0.275
